# Web-based electronic health record system

**DOI:** 10.1186/1472-6963-12-S1-P1

**Published:** 2012-11-21

**Authors:** Aniruddha Patil, Pranav Manikpure, Shankar Kokare, Makarand Nale, MS Chaudhari

**Affiliations:** 1 Dept. of Computer Eng., SIT, Pune University, Lonavala 410401, India

## Introduction

The use of healthcare delivery systems is very limited. Primarily in India public sector hospitals does not use any system for managing their health records to provide continued care to patient. The central and state governments are responsible for the provision of primary healthcare in the country. A spending of 1% of the GDP (effectively about Rs 1050 per capita) on public health is not only dismally low but most of the expenditure is on staff salaries leaving little or nothing for facilities, drugs and other consumables.

## Methodology

During the registration process patient’s previous medical history, the data to be stored is divided in two sections as administrative and clinical content which is as follows: the administrative content includes: (1) identification – patient’s complete name, medical record number, address, mother’s maiden name; (2) lifestyle indicators – education level, profession, allergies, chronic illnesses, marital status, food, preferences, smoking and alcohol consumption. The clinical content includes: (1) symptoms, physical examination results; (2) drugs prescribed, inpatient history; (3) lab reports - pathology/ radiology/ ECG / EEG / EMG.

## Results

The EHR system: (1) must be capable of containing information on: healthcare processes, activities, medical problem, healthcare requests, healthcare characteristics, resources, users and authorization. (2) Should allow for the recording of all data on the patient history, physical examination, diagnostic test, and therapeutic interventions to support patient care. (3) Should allow for pre-birth and post-death entries. (4) Should allow for the recording of interpretations, observations, decisions request for further investigations, treatment or discharge. (5) Must be able to reflect that a patient may have concurrent problems. (6) Should support the use of technologies, decision support and management plans.

## Conclusion

The software we are going to design definitely more helpful to the doctor and patient. The complexity of the paper work will be reduced and provide the future health support to the patient and the country. Benefit of the software is:(1) healthcare awareness of patient increases, (2) demographic information will be available for planning better healthcare delivery, (3) addresses the issue of patient mobility as patient information is available for all healthcare centers as it is web enabled. By deploying such software in different healthcare centers in rural India, we can increase the usage of EHRs thereby increasing the efficiency of Healthcare Delivery and reducing the cost of healthcare.

